# Magnetic stimulation of the angiogenic potential of mesenchymal stromal cells in vascular tissue engineering

**DOI:** 10.1080/14686996.2021.1927834

**Published:** 2021-06-28

**Authors:** Ana C. Manjua, Joaquim M. S. Cabral, Carla A. M. Portugal, Frederico Castelo Ferreira

**Affiliations:** a LAQV-REQUIMTE, Departamento de Química, NOVA School of Science and Technology, Universidade Nova de Lisboa, Caparica, Portugal; b Department of Bioengineering and iBB – Institute for Bioengineering and Biosciences, Instituto Superior Técnico, Universidade de Lisboa, Lisboa, Portugal

**Keywords:** Magnetic field, scaffolds, MSCs, VEGF, angiogenesis, 30 Bio-inspired and biomedical materials, 211 Scaffold / Tissue engineering/Drug delivery, 212 Surface and interfaces

## Abstract

The growing prevalence of vascular diseases worldwide has emphasized the need for novel tissue-engineered options concerning the development of vascularized 3D constructs. This study reports, for the first time, the use of external magnetic fields to stimulate mesenchymal stromal cells (MSCs) to increase the production of vascular endothelial growth factor-A (VEGF-A). Polyvinylalcohol and gelatin-based scaffolds, containing iron oxide nanoparticles, were designed for optimal cell magnetic stimulation. While the application of static magnetic fields over 24 h did not impact on MSCs proliferation, viability and phenotypic identity, it significantly increased the production of VEGF-A and guided MSCs morphology and alignment. The ability to enhance MSCs angiogenic potential was demonstrated by the increase in the number of new vessels formed in the presence of MSCs conditioned media through *in vitro* and *in vivo* models. Ultimately, this study uncovers the potential to manipulate cellular processes through short-term magnetic stimulation.

## Graphical abstract

**Figure:**
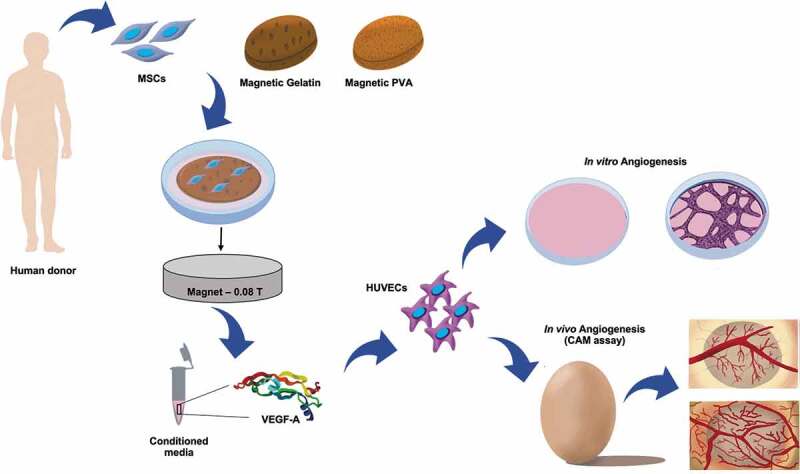

## 1. Introduction

Therapeutic angiogenesis, involving the delivery of angiogenic factors to ischemic tissues to promote blood vessel formation and restore blood flow, has been suggested two decades ago [1]. Yet vascular diseases, especially cardiovascular, are still recognized as a major cause of mortality and morbidity, causing more than 30% of global deaths every year [2,3]. In the past years, and despite the primary treatment with pharmacological agents, surgery or endovascular intervention, no better treatment has become available for the patients who have not achieved a complete revascularization or are not suitable for revascularization [1,4].

Novel strategies in tissue engineering requiring the ability to promote adequate vascularization for tissue substitutes are therefore a critical and yet unmet need these days. The main challenge in this field remains the mass transfer hindrances, limiting the size of any tissue constructs grown *in vitro* and the subsequent integration of these constructs *in vivo* [5]. Additional strategies for enhancing vascularization and ensuring the survival of large tissue-engineered grafts include scaffold design, the inclusion of angiogenic factors and both *in vivo* and *in vitro* pre-vascularization [6,7].

Mesenchymal stromal cells (MSCs) have also become scientifically interesting given the variety of bioactive molecules they release when properly stimulated. The MSC and its secretome have the potential for clinical translation. The secretome of MSCs includes several cytokines and chemokines, some of which are important mediators of MSCs homing effect; growth factors and pro-angiogenic molecules (e.g. VEGF, PDGF, TGF-ß, FGFs, among others); and anti-inflammatory factors (e.g. iNOS, IL-6, HGH, and others) able of immunomodulatory properties. These signaling molecules are presented as soluble factors or transported on extracellular vesicles [8–11].

VEGF-A, a potent angiogenic factor and often released as a cell-survival signal, is one of the most important paracrine factors involved in the regulation of the interactions between MSCs and endothelial cells leading to formation of microvessel-like structures [4,8,12]. This molecule has been exhaustively studied as a target molecule to stimulate or inhibit angiogenic phenomena [4,8,12,13]. Some papers have reported how the induced mobilization of VEGF from bone marrow-derived endothelial progenitor cells is able to potentiate cells differentiation *in vitro* as well as trigger neovascularization *in vivo* [4,14]. Other studies demonstrated that MSCs are capable of inhibiting endothelial proliferation and angiogenesis through cell-cell contact and modulation of the VE-cadherin/ß-Catenin signaling pathways [15]. Still a powerful challenge in this emerging field involves the development of a controlled system to stimulate the secretome of MSCs into releasing cell-survival signals to promote the formation of microvessel-like structures.

Although inconsistent toxic effects of static magnetic fields (in the range of 0.5–5 T) on different cell types have been reported over the years [16–18], some recent works confirmed a potential benefit in using magnetic stimulation over cell fate regulation shifting towards mechanical stimulation and induction of mechanotransduction phenomena in the process. Most of these works highlight the effect of the magnetic forces (5 mT-0.1 T) on promoting cell differentiation in *in vitro* models or even to enhance bone repair *in vivo* [19–21]. Interestingly, a neuronal model of ischemia/reperfusion (I/R) injury confirmed the neurobiological mechanisms of frequency-dependent repetitive magnetic stimulation in ischemia/reperfusion injury-treated neuronal cells by activating extracellular signal-regulated kinases and AKT-signaling pathway and thus increasing cell proliferation and inhibiting apoptosis in injured cells [22]. Moreover, magnetically responsive hydrogels of *k*-carrageenan doped with magnetic nanoparticles have been used to modulate chondrogenic differentiation in adipose-derived stem cells; dextran grafter maghemite nanoarchitectures, integrated with chitosan-based scaffolds, have been reported to improve proliferation of both osteosarcoma cell line (MG63) and human mesenchymal stem cells; and poly(ϵ-caprolactone)/iron-doped hydroxyapatite scaffolds were shown to promote cell viability, adhesion and proliferation and osteogenesis *in vitro* [23–25]. Finally, static magnetic field (24 mT) has been reported to significantly decrease MSCs proliferation [26].

The current study aims to investigate whether non-invasive magnetic stimulation can address the unmet challenge to promote vascularization, overcoming tissue dimension limitations. Hence, the effects of applying a remote static magnetic field (alone or in combination with magnetic responsive scaffolds) to stimulate VEGF secretion by bone marrow-derived MSCs, and subsequent formation of microvessel-like structures from human umbilical vein endothelial cells (HUVECs) are discussed in this paper. The study includes:

1. the development and characterization of polyvinylalcohol (PVA) and gelatin hydrogels, doped with iron oxide nanoparticles (MNPs), hereafter named mGelatin and mPVA, respectively;
2. the evaluation of the impact of the magnetic forces on the proliferation, viability, distribution and phenotypic identity of the MSCs cultivated in 2D or 3D, first on standard tissue culture plates (TCP) and then on magnetic responsive scaffolds (mPVA and mGelatin);
3. the analysis of expression and quantification of VEGF-A produced and secreted by MSCs, upon seeding on both mPVA) and Gelatin (mGelatin) scaffolds integrating dispersed MNPs, and under exposure to static magnetic field; and
4. further investigate the potential effect of the magnetic field on the formation of new microvessels, *in vitro* and *ex vivo*, as well as the impact on enhancing *in vitro* wound healing and MSC migration.

Ultimately, this work aims to highlight the potential of using magnetic stimulation and mPVA and mGelatin scaffolds to modulate cell fate and behavior, namely exploring the impact of magnetically stimulated MSCs secretome on the formation of new microvessels. With this approach, we hope to open new possible therapeutic approaches for blood vessels regeneration with potential clinical translation to treat several vascular diseases.

## 2. Experimental section

### 2.1. Scaffold fabrication

Magnetic nanoparticles of iron oxide (MNPs) were synthesized by chemical co-precipitation of iron salts FeCl_3_ and FeCl_2_ (Sigma-Aldrich, U.S.) in alkaline media. The MNPs used with gelatin were synthesized according to the protocol previously published by Izquierdo et al. [27]. An aqueous solution with 25% of ammonium hydroxide (NH_4_OH) (Fluka, Germany) was added to a mixture of FeCl_3_ and FeCl_2_ at 80°C, under permanent stirring at 1250 rpm in N_2_ atmosphere. The MNPs used in combination with PVA were synthesized according to the protocol previously published by Olle et al. [28]. In this case, potassium oleate and ammonium persulfate (Sigma-Aldrich) were then added to produce Fe_3_O_4_-coated particles to minimize aggregation and Hitenol-BC (Sigma-Aldrich) worked as a surfactant.

Porcine skin gelatin (8% m/v, type A, G2500, Sigma-Aldrich) and PVA (10% m/v, Mowiol 10849, Sigma-Aldrich) were dissolved in milli-Q water (10% m/v) at 60°C in two different preparations. In both situations, MNPs were dispersed by sonication (3 h, 60°C) in the polymeric solutions. For PVA, (Sigma-Aldrich, >99%), 3% of glutaraldehyde (cross-linking agent, 25% in H_2_O, Sigma-Aldrich) and 3% of HCl (37%, Sigma-Aldrich) were sequentially added to the solution before casting onto glass plates. The gelatin matrices were cast onto glass plates and left overnight at 4°C. The samples were removed from the glass plates and immersed in an aqueous solution containing 1% of glutaraldehyde (25% in H_2_O) for 3 h. The magnetic hydrogels were then washed by immersion in a demineralized water bath. The procedure was followed by optical absorption measurements overtime (230–930 nm). For sterilization, the samples were cut according with the shape and size of the 24-well cell culture plates (TCP, Corning®, New York, U.S.) and immersed in a PBS solution containing 1% of antibiotic-antimycotic (Gibco, U.S.). For better conditioning of the cells, the samples were immersed in cell culture media for 3 h before seeding the cells on top of the hydrogels.

### 2.2. MNPs and magnetic responsive scaffold characterization

The MNP size distribution was assessed by transmission electron microscopy (TEM) using aTEM Hitachi H8100 (LaB_6_ filament and an acceleration tension of 200 kV, Tokyo, Japan) . The MNP sizes were determined using ImageJ software and the average particle size was obtained by statistical analysis of a total of 320 counts (95% confidence). Dynamic light scattering technique (DLS, Malvern Zetasizer Nano ZS, UK) was also used to obtain information on the nanoparticle size and aggregation using a refraction index of 2.4 for iron oxide nanoparticles (Fig. S1a). These MNPs are then dispersed in the hydrogels of PVA and Gelatin, leading to magnetic scaffolds of mPVA and mGelatin. The dispersion quality of the MNPs in the hydrogels was inspected by optical microscopy using a microscope (Olympus BH-2, Tokyo, Japan) set with a photo camera (Casio Exilim Pro, EX-F1, Tokyo, Japan). Surface roughness was obtained by confocal microscopy using a confocal laser scanning microscope (LSM 700/Carl Zeiss, Germany) for 3D imaging of the surfaces and image analysis was obtained using ZEN 2.1 software (Carl Zeiss).

Surface contact angles of the scaffolds were determined using glycerol as the liquid phase in the absence and presence of a magnetic field up to 0.08 T. The magnetic field was created by a neodymium magnet placed underneath the hydrogel scaffolds. The dynamic glycerol contact angles were determined in a sessile drop mode using a drop shape analyzer system coupled to a video camera connected to a PC for data acquisition. The average contact angles values were obtained for at least three triplicates. The magnetic characterization of the hydrogels was performed using Vibrating Sample Magnetometer (VSM), Physical Property Measurements System from Quantum Design, Inc., at UNIZAR (Zaragoza, Spain). Magnetization saturation values were recorded for the scaffold with MNPs at 300 K in applied magnetic field of up to 1.2 T (Fig. S1b).

### 2.3. Cell culture

Human bone-marrow MSCs lines used in this paper are part of the cell bank available at Stem Cell Engineering Research Group (SCERG), iBB-Institute for Bioengineering and Biosciences at Instituto Superior Técnico (IST). Bone marrow aspirates were obtained from Instituto Português de Oncologia Francisco Gentil, Lisboa–Portugal, under collaboration with iBB-IST. All human samples were obtained from healthy donor after written informed consent, in accordance with the Directive 2004/23/EC of the European Parliament and of the Council of 31 March 2004 on setting standards of quality and safety for the donation, procurement, testing, processing, preservation, storage, and distribution of human tissues and cells (Portuguese Law 22/29 June 2007), with the approval of the Ethics Committee of the respective clinical institutions [29]. Isolated cells were cryopreserved in liquid/vapor nitrogen tanks until further use. Isolated human bone marrow MSCs (BM MSCs) were cultured using low-glucose Dulbecco’s Modified Eagle Medium (DMEM, Gibco, Grand Island, New York, U.S.) supplemented with 10% fetal bovine serum (FBS MSC qualified, Gibco) and 1% antibiotic-antimycotic (Gibco) and kept at 37°C, 5% CO_2_ and 21% O_2_ in a humidified atmosphere. Three independent donors, with ages ranging between 33 and 68 years old, were used in the experiments (n = 3). HUVECs were purchased from Lonza (Basel, Switzerland) and maintained in commercial endothelial growth medium-2 (EGM-2, Lonza) and kept at 37°C, 5% CO_2_ in a humidified atmosphere. Medium renewal was performed every 3–4 days. All the experiments were performed using cells between passages 4 and 7.

MSCs were seeded on the scaffolds at a density of 75,000 cell/cm^3^ using expansion medium (DMEM + 10% FBS) on cell culture plates with a covalently bound hydrogel layer to inhibit cellular attachment (Costar® 24 ultra-low attachment well plates). As a control, MSCs were seeded as a monolayer (2D cultures) on cell culture plates (TCP, Corning®) at a density of 75,000 cell/cm^2^. MSCs were allowed to adhere to the supporting material (TCP or scaffolds) for 24 h at 37°C in humidified atmosphere, before magnetic exposure.

3D culture featuring MSC spheroids was obtained using the hanging drop technique. According to the technique, several drops 10 µL of cell culture media, containing 1,000 cells/µL, were placed on the top part of a petri dish (Fisher Scientific) and incubated for 24 h to force cell aggregation at 37°C in a humidified atmosphere. The method resulted in spheroids with 10,000 cells, which were carefully collected using the tip of a 20 µL micropipette and placed in culture using expansion medium in ultra-low attachment well plates (Costar® 24 ultra-low attachment well plates) for another 24 h before magnetic stimulation.

Magnetic stimulation was performed by placing a circular neodymium magnet on the second day of the cell culture, beneath the cell culture plates containing MSCs (TCP), under 2D or 3D cultures or growing on scaffolds (mPVA, mGelatin) for a total period of 24 h and allowing a maximum intensity of 0.08 T in the cell culture. This step requires precision to ensure the entire area of the cell culture wells is covered by the magnet. Prior to the exposure, collected powder MNPs were used to determine the direction of the magnetic field and a gaussmeter was used to determine the magnetic intensity sensed by MSCs (0.08 T).

### 2.4. Human bone marrow mesenchymal stromal cells (BM MSC) metabolic activity and proliferation curves

The metabolic activity of MSCs was evaluated using AlamarBlue® cell viability reagent (Molecular probes, Eugene, Oregon, U.S.), on days 1, 3, and 5 of the cell cultures. AlamarBlue® cell viability reagent was added to the cells and incubated at 37°C in 5% CO_2_ chamber for 2 h. Fluorescence was quantified at a wavelength range of 560–590 nm. Prior to analysis, a calibration curve for different MSC densities (10,000, 20,000, 50,000, 75,000, 100,000, 150,000 cells/mL) was obtained relating cells counting to the metabolic activity of the MSCs measured by AlamarBlue® cell viability reagent, following manufacturer instructions. The calibration curve was used to convert the obtained metabolic values into cell numbers and determine the proliferation curves associated with each condition. All conditions were tested on triplicates using three different donors (donors 1, 2 and 3).

### 2.5. Cell viability and morphology assay

MSCs viability and morphology (cultured in 2D- 3D cultures and in scaffolds of mPVA and mGelatin) were assessed on experimental day 1, after magnetic exposure. Cells were washed twice with phosphate buffer saline (PBS, Dulbecco’s Sigma-Aldrich), and stained using LIVE/DEAD^TM^ Viability/Cytotoxicity Kit, for mammalian cells (ThermoFisher Scientific, L3224) according to the manufacturer protocol, for 20 min. MSCs were imaged in a fluorescence microscope (Leica DM IL LED with EC3 camera system, Germany), and afterwards washed again twice with PBS and fixed with 4% paraformaldehyde (Sigma-Aldrich) for 20 min. Cells were permeabilized with 0.1% Triton X-100 for 10 min and incubated with Phalloidin-TRITC (Invitrogen, 1.5 µg/mL) or Alexa Fluor 488 (ThermoFisher, 4 µg/mL) for 20 min and 4′,6-diamidino-2-phenylindole (DAPI, ThermoFisher Scientific, 1.5 µg/mL) for 5 min, then washed with PBS for fluorescence imaging.

### 2.6. Metabolite analysis

Glucose and lactate concentrations were determined in the conditioned media of the samples collected from MSCs overtime using an automatic analyzer (YSI 7100MBS; Yellow Spring Instruments, U.S.).

### 2.7. Immunophenotype characterization of magnetically stimulated MSCs

For immunophenotypic characterization, cells were tested by flow cytometry after 5 days, with and without magnetic exposure, for expression of cell surface markers indicative of MSC using a panel of mouse anti-human monoclonal antibodies (PE-conjugated) against: CD73^+^, CD90^+^, CD105^+^, CD14^−^, and human leukocyte antigen HLA-DR^−^ (all from Biolegend, California, U.S.). The cells were incubated with the monoclonal antibodies for 15 min in the dark at room temperature. Then, cells were washed with PBS and fixed with 4% paraformaldehyde (Sigma-Aldrich). Appropriate isotype controls (IgGy1 and IgGy2b) were also prepared. A minimum of 7,000 events were collected for each sample, and Cell Quest (Becton Dickinson, New Jersey, U.S.) and FlowJo® (LLC) software were used for acquisition and analysis, respectively.

### 2.8. Cytotoxicity assay

The biocompatibility of the scaffold (mPVA and mGelatin) was demonstrated through a cytotoxicity assay. Fibroblasts (L929 mouse line) were seeded at 80,000 cells/cm^2^ in a 24 well plate and incubated for 48 h with low-glucose Dulbecco’s Modified Eagle Medium (DMEM, Gibco, Grand Island, New York, U.S.) supplemented with 10% fetal bovine serum (Gibco) and 1% antibiotic-antimycotic (Gibco) and kept at 37°C, 5% CO_2_ and 21% O_2_ in a humidified atmosphere. Sterile scaffolds were placed on top of the fibroblasts monolayer for 24 h and then observed in the optical microscope for quantification of the halo formed in the area between the scaffold and the monolayer. Indirect assay was also performed using latex material as the positive control and fibroblasts culture media as the negative control. The lixiviates of the scaffolds incubated for 48 h to allow release of eventual toxic substances to the media were used as a replacement for the fibroblasts culture media and incubated for another 24 h. MTT solution (1 mg/mL) was prepared and replaced the lixiviates in the fibroblasts culture, in a 2 h incubation period. MTT solvent (HCl and IPA – 1:100, Sigma-Aldrich) is added to MTT solution in the cell culture and stirred for 5 min. Absorbance is quantified at 570 nm to determine total cell viability.

### 2.9. Quantitative reverse transcription-polymerase chain reaction analysis

The expression levels of key angiogenesis factor VEGF-A genes were quantified from the extraction of the RNA of MSCs for all the experimental groups (2D-3D cultures, cultures on scaffolds of mPVA and mGelatin). MSCs were detached (either from scaffolds or TCP) and lysed, after 24 h of magnetic exposure, using consistent up and down pipetting movements for 10 min and a lysis buffer (RLT), which is part of the RNeasy Mini Kit (Qiagen, Hilden, Germany) for RNA extraction. The detachment process was aided by placing the culture plates in a stirring plate (300 rpm) for 10 minutes. Cell lysates were stored at −80°C.

Subsequent procedures for total RNA extraction were performed following the manufacturer’s instructions on the RNeasy Mini Kit. Complementary DNA was synthesized from 20 nm of total RNA using iScript Reverse Transcription Supermix (Bio-Rad, Hercules, California, U.S.). Reaction mixture (20 µL) was incubated in a 96-well thermal cycler (Applied Biosystems, Foster City, California, U.S.) for 5 minutes at 25°C, 30 minutes at 42°C and 5 minutes at 85°C and then maintained at 4°C. Gene expression levels of VEGF-A were assessed. Sequences of the specific primer sets are given in Table S1, Supplementary Information. The quantitative reverse transcription-polymerase chain reaction (qRT-PCR) was performed using SYBR Green PCR Master Mix (Applied Biosystems). All reactions were carried out at 95°C for 10 minutes, followed by 40 cycles of 95°C for 15 seconds and 60°C for 1 minute, according to manufacturer’s instructions. Glyceraldehyde 3-phosphate dehydrogenase (GAPDH) was used as internal control to normalize differences in total RNA levels in each sample. A threshold cycle (C*t*) was observed in the exponential phase of amplification, and quantification of relative expression levels was performed using standard curves for target genes and endogenous control. Geometric means were used to calculate the ΔΔC*t* values and are expressed as 2^−ΔΔC*t*^. The mean values from triplicate analysis were compared. All conditions were tested on triplicates using three different donors (donors 1, 2 and 3).

### 2.10. Quantification of VEGF secretion by enzyme-linked immunosorbent assay (ELISA)

For quantification of the total amount of exogenous angiogenic VEGF-A protein present in MSCs conditioned media in all groups of analysis, an ELISA experiment was performed. To quantify ELISA, culture supernatant was collected at each time point (24 h) for MSCs in 2D-3D cultures or cultivated on scaffolds (mPVA and mGelatin) and exposed or not to the magnetic field (0 T and 0.08 T). The conditioned media was kept at −80°C until further analysis. Human VEGF-A kit (RayBiotech, U.S.) was performed following the manufacturer’s instructions. All conditions were tested on triplicates using three different donors (donors 1, 2 and 3).

### 2.11. In vitro *endothelial cell tube formation assay*

A functional assay to assess the capacity of HUVECs to form microvessels upon culture on a Matrigel substrate (Corning®, New York, U.S.) was used to evaluate the angiogenic potential of the magnetic system. The effect of the exogenous VEGF-A present in the conditioned media on the conditions of analysis was evaluated in a 3D capillary-like tube formation assay. Briefly, HUVECs (2x10^4^ cells/cm^2^) were cultured on Matrigel (50 µL/well) in a 96-well plate (Corning® 96-well microplates) and supplemented with the conditioned media from MSCs in 2D cultures or cultivated on scaffolds (mPVA and mGelatin). The conditioned media was collected after 24 h of exposure to the magnetic field (0.08 T) or without magnetic exposure (0 T). After incubation for 6 h at 37°C, photographs of the center of the well were taken using a light microscope (Leica DM IL LED with EC3 camera system) and the number of tubular-like structures and branch points formed were counted using ImageJ (NIH) software with a 95% of confidence level. For this experiment, independent experiments of magnetic stimulation were performed where MSCs were incubated with Endothelial Cell Basal Medium (EBM-2, Lonza) throughout the experiment. All conditions were tested on triplicates using three different donors (donors 1, 2 and 3).

### 2.12. Wound healing and MSCs migration assay

A functional assay to assess wound healing was developed using silicone inserts featuring two chambers with a maximum volume capacity of 70 µL and a compartment-gap width of 500 µm (Culture-insert 2-well 35 mm, IBIDI®, U.S.). After inserting the constructs on 24-well plates (TCP, Corning®), HUVECs (2x10^4^ cells/mL) were then cultured in each chamber until reaching confluency. The construct was then carefully removed leaving a gap between the cells from each chamber. HUVECs were treated with conditioned media from MSCs exposed to magnetic field for 24 h (0.08 T) and a control for MSCs not exposed to magnetic forces (0 T). The gap area was measured overtime through images of the same region. A second independent experiment was carried out to evidence the direct impact of the magnetic field on the ability of the MSCs to migrate and close the wound. MSCs were cultured on 24-well plates (TCP, Corning®) and allowed to reach confluency. A micropipette (20 µL tip) was then used to scratch the monolayer of confluent MSCs and create a gap in the MSCs monolayer. From this moment onwards, a magnet (0.08 T) was placed underneath the wells and images were taken overtime to monitor the closing of the gap. A control group with no magnetic exposure (0 T) was also assessed over time to compare the performance of both groups. All conditions were tested on triplicates using two different donors (donor 2 and 3).

### 2.13. Chick chorioallantoic membrane (CAM) assay and neo-vessel quantification

The angiogenic potential of the secretome of MSCs exposed to a magnetic field for 24 h was evaluated using the CAM assay under the following analysis conditions: MSCs cultured on gelatin scaffold (0.08 T and 0 T); 2D cultures of MSCs (0.08 T and 0 T), as controls. Briefly, fertilized chick (*Gallus gallus*) eggs were horizontally incubated at 37°C in a humidified atmosphere. Three days after the embryonic day, 1.5–2 mL of albumin was removed to allow the detachment of the developing CAM and a square incision was made on the eggshell under sterile conditions. The window was sealed with sterile transparent adhesive tape and the eggs returned to the incubator. On day 10-post fertilization, a 3 mm silicone ring was placed on top of the CAM and 10 µL of the conditioned media to test were added to the CAM of the chicken embryo (n = 6–10 per experimental group, using two different donors: 2 and 3). To avoid additional variability, one pair of conditions (the secretome under magnetic exposure: 0.08 T, and the respective control: 0 T) was combined in the same egg and individually placed on different sections of the chicken embryo. Sterile parafilm tape was used to seal back the window and the eggs were further incubated for 3 days, being hydrated every day. Gestational process was terminated with the embryos being euthanized with fixative (2 mL) on top of the CAM and the ring was removed for the CAM to be excised. The inoculation area was then observed under a stereoscope, at 20x magnification (Olympus, SZX16, coupled with a DP71 camera) [30,31]. The number of new vessels, lower than 20 µm diameter, growing radial towards the ring area is registered. All animal procedures were carried out in accordance with the guidelines of the European Directive 2010/63/EU and the national Decreto-Lei nº 113/2013.

### 2.14. Statistical analysis

All measurements were made at least three times, in independent conditions. All results are shown as the mean ± standard deviation (SD). Two-way ANOVA following Sidak’s multiple comparisons test was used to compare the mean of three values obtained from three independent conditions for each donor in a total of 3 donors (n = 3). This statistical analysis was applied to RT-PCR, ELISA and *in vitro* sprouting experiments, using GraphPad Prism version 7 (GraphPad Software, La Jolla, CA, U.S.). The parametric student’s *t* test, two-sided, was performed to determine statistically significant differences on *in vitro* wound healing and *ex vivo* CAM assay experiments; *p < 0.05 indicates a significant result; **p < 0.01 a very significant result, ***p < 0.001 a highly significant result, and ****p < 0.0001 an extremely significant result.

## 3. Results

### 3.1. Scaffolds magneto-responsiveness are modulated by MNPs load and magnetic exposure

Two magnetic responsive scaffolds were fabricated:

(i) the mPVA hydrogel, made of PVA, a synthetic material, doped with magnetic nanoparticles (mPVA), was made as previously described and used to control protein sorption [32], and (ii) a newly developed magnetic scaffold based on biodegradable denatured collagen (gelatin), a natural polymer suitable to promote cell adhesion in tissue culture [33], with embedded MNPs, mGelatin.

**Figure 1.**
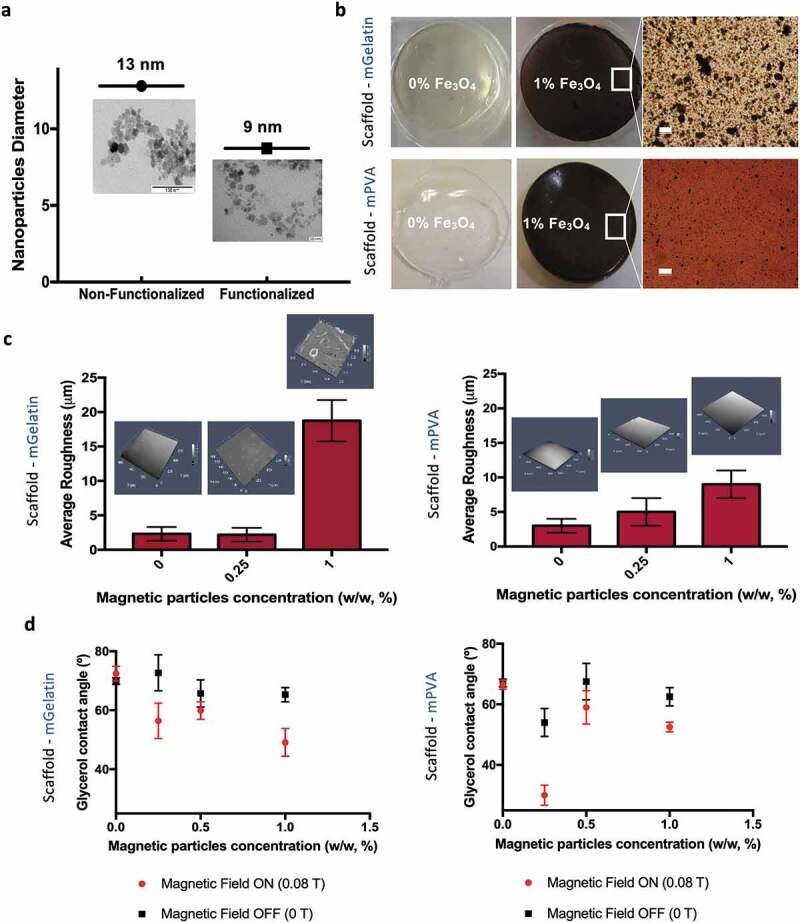
The surface properties of magnetic gelatin (mGelatin) and magnetic PVA (mPVA) is dependent on the magnetic load. (a) Functionalized MNPs were used to fabricate mPVA while non-functionalized MNPs, to induce the formation of magnetic clusters, were used on Gelatin (mGelatin). Representative TEM images to determine MNPs average diameter. (b) Images of the scaffolds with and without addition of MNPs in their composition. Scale bar: 100 µm. (c) Quantification of the average roughness of the scaffolds with different magnetic loads was assessed through 3D confocal imaging of the scaffolds surface, showing roughness increase with the magnetic concentration. (d) Determination of the contact angle observed in mPVA and mGelatin scaffolds, according with the magnetic load and the presence of a magnetic field of low intensity (0.08 T). mPVA results: Reprinted (adapted) with permission from Manjua, A. C., Alves, V. D., Crespo, J. G. & Portugal, C. A. M. Magnetic Responsive PVA Hydrogels for Remote Modulation of Protein Sorption. ACS Appl. Mater. Interfaces 2019, 11, 23, 21239–21249 (2019). Copyright (2019) American Chemical Society

Nanoparticles integrated on the PVA scaffold were prepared by chemical functionalization to avoid aggregation, and their size ranged from 2 to 20 nm with an average diameter of ~9 nm (Figure 1(a)). Non-functionalized nanoparticles were determined with sizes ranging from 6 to 21 nm (average diameter ~13 nm) (Figure 1(a)). Due to the increased aggregation tendency of non-functionalized MNPs, these were incorporated into gelatin-based scaffolds (mGelatin) to investigate the possibility of achieving enhanced magnetic response. The hydrogels obtained were characterized in terms of MNPs dispersion quality by optical microscopy, with evident nanoparticle aggregation observed in the mGelatin and a more uniform MNPs dispersion in the mPVA hydrogel (Figure 1(b)). Similarly to the stability of mPVA, which has been previously demonstrated [32], the stability of mGelatin scaffold was confirmed by optical absorption. No release of MNPs to the media was detected in these experiments (Supplementary Fig. S2a). The swelling of the hydrogels was also determined, with mGelatin evidencing a higher swelling degree in comparison with mPVA (Supplementary Fig. S2b).

The mPVA average surface’s roughness increased, up to nearly 10 µm in roughness, with the increase of MNPs concentration (0%, 0.25% and 1%). Low average roughness was also obtained for mGelatin containing lower MNP loads (0% and 0.25%), however mGelatin scaffold roughness steeply increased to the higher MNPs load (1%) used, reaching an average roughness of 18 µm (Figure 1(c)). As previously reported for the mPVA hydrogels [32], an increase in the surface hydrophilicity was also observed for the mGelatin hydrogels as a response to magnetic exposure, perceived through a 20° decreased of the surface contact angle of the mGelatin hydrogels prepared with the highest MNPs loads (Figure 1(d)).

### 3.2. Magnetic forces impact on 2D and 3D cultures of MSCs

**Figure 2.**
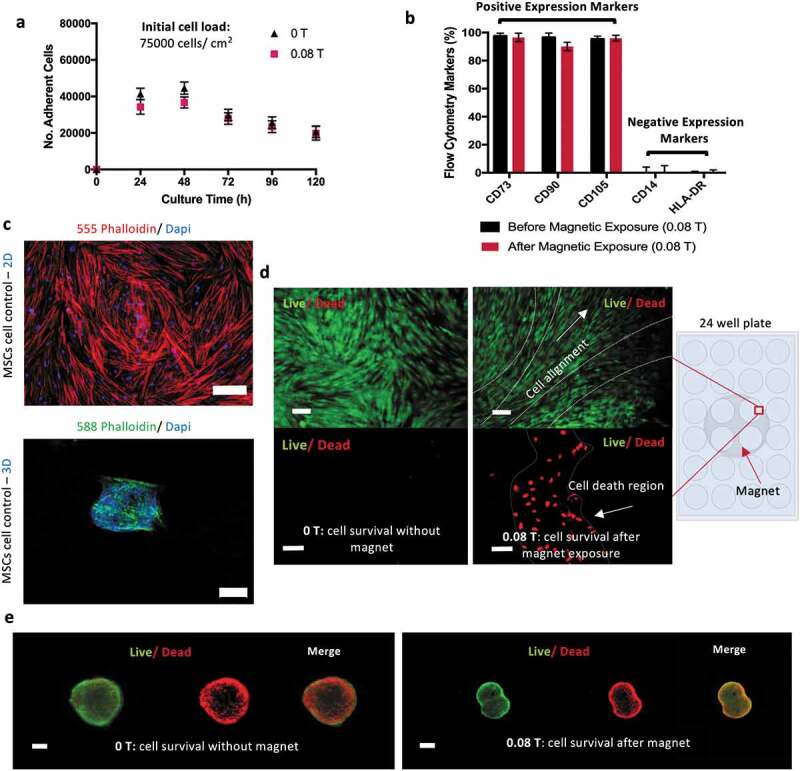
Magnetic effect in a 2D culture of MSCs reflects on cell alignment while 3D cultures do not show magnetic sensitivity. (a) Adherent MSCs cultured on cell culture plate under magnetic field (0.08 T) against control conditions (0 T) showed that the magnetic field does not impact on cell growth. The metabolic activity of the cells was obtained using AlamarBlue staining proliferation assay. (b) Representation of the expression of specific MSCs positive and negative markers by flow cytometry suggests the magnetic field does not modify the phenotype of the cells after 5 days of continuous exposure. (c) Confocal images of the morphology of MSCs monolayer stained with 555 phalloidin (red) and DAPI (blue) and MSCs in 3D stained with 588 phalloidin (green) and DAPI (blue). Scale bar: 100 µm. (d) Live (Green)/Dead (Red) images of MSCs aligning in accordance with magnetic forces. Scale bar: 100 µm. (e) 3D cultures of MSCs do not show changes upon magnetic field exposure. Data presented as means ± SD

Cell culture on monolayer (2D) was performed first on standard polystyrene tissue culture plates (TCP), in the absence and in the presence of the magnetic field. This study allows us to investigate the impact of the magnetic field on the MSCs without effects driven by the interactions between cell and scaffolds. MSCs proliferation and immunophenotype profile were characterized. The number of MSCs increased steeply on the first days, reaching confluency after 48 h, with a total amount of 50,000 cells/mL. Cells number started then to slowly decrease until the end of the experiment (96 hours). Notably the magnetic field does not seem to impact on the proliferation curves, which show the same behavior over time in the presence and absence of the magnetic field at a value of 0.08 T (Figure 2(a)).

Metabolite analysis (glucose and lactate) confirmed that glucose and lactate concentrations remain, respectively, above the critical value of 1 mM and below toxic threshold of 10 mM, thus indicating a healthy glucose/lactate balance in the culture, without carbon source limitations (Supplementary Table S2). MSCs identity phenotype was assessed before and after 5 days of continuous exposure to the magnet. Flow cytometry showed positive (CD73, CD90, CD105) and negative (CD14, HLA-DR) expression of the surface cell markers. No statistical significance was obtained for these results, suggesting that the exposure to a 0.08 T magnetic field does not affect MSC phenotype (Figure 2(b)).

MSCs were also cultivated in 3D as cellular aggregates (spheroids), in the presence and absence of magnetic field, as a 3D cell model, and cells morphology and viability were compared with MSC cultured on monolayer. 2D and 3D cultures were observed by confocal microscopy imaging (Figure 2(c)) showing, as expected, the characteristic stretched cell shape for 2D monolayer and the cell clustering morphology for 3D cultures. Despite the high number of living cells in both, the control (in the absence of magnetic field) and the magnetic exposed conditions, the Life/Dead assay images (Figure 2(d-f)) suggested some cell death in 2D cultures during the magnetic induced cell alignment. Regarding the 3D cell culture, no relevant differences were observed by the exposure to the magnetic field, possibly due to the capacity of these structures to move within the media diminishing the prolonged impact of the static magnetic field over these spheroids (Figure 2(e)). However, a necrotic core was observed over time in MSCs spheroids (200–400 µm), with or without magnetic stimulation, which can be associated with oxygen and nutrients depletion and accumulation of waste products in their core. This effect has been described in the literature before, limiting cell spheroids maximum diameter, depending on cell type [34–36].

### 3.3. MSCs geometry and alignment in scaffolds are magnetic field-dependent

The interactions of cells, scaffolds and magnetic fields are here studied. The potential cytotoxicity of MNPs, placed within the scaffold, was assessed through ISO 10993 using fibroblasts. The contact assay showed no formation of inhibition halo in the contact area between the scaffolds and the fibroblasts layer, suggesting the biocompatibility of these magnetic scaffolds for MSCs culture (Supplementary Fig. S3a). Indirect contact measurement using MTT assay confirmed the biocompatibility by revealing a cell viability over 90% for scaffolds doped with MNPs (Supplementary Fig. S3b), which is consistent with our results where no released MNPs were detected.

**Figure 3.**
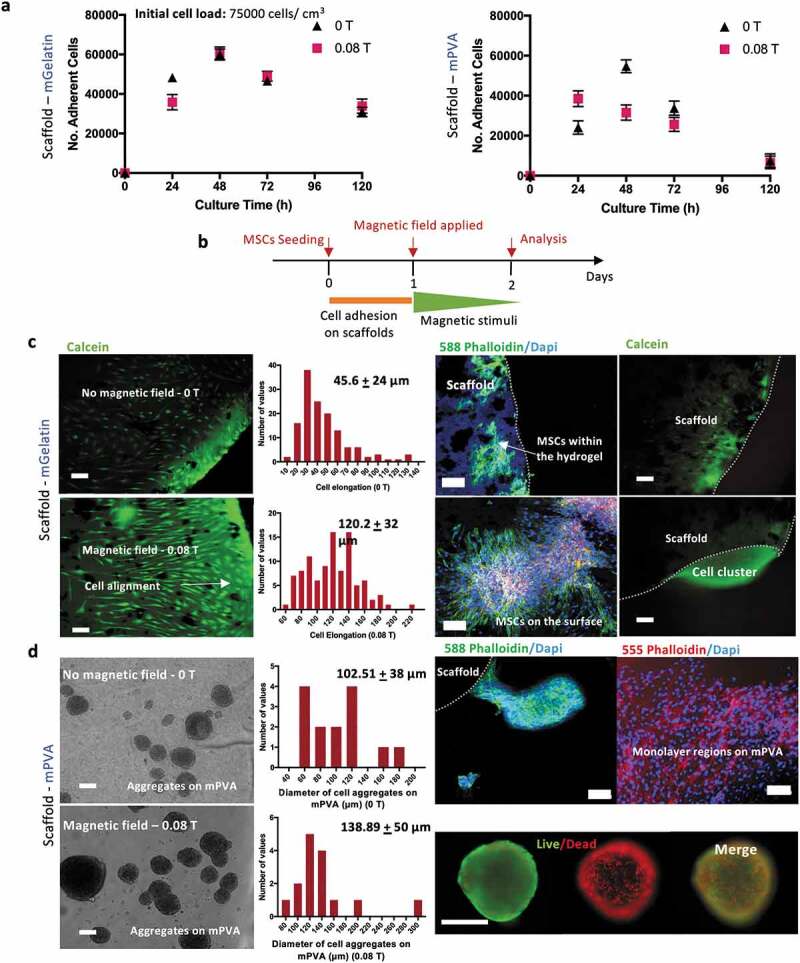
MSCs interaction with magnetic scaffolds reveals morphologic impact depending on the nature of the scaffold and the magnetic exposure. (a) Adherent MSCs cultured on mPVA and mGelatin overtime showed an adhesion efficiency of 47–67% for the mGelatin and 33%-50% for the mPVA. The magnetic field indicated a slight increase in proliferation in the mPVA but no differences were observed for the mGelatin. The long-term survival of the cells is suggested to be independent of the magnetic exposure. (b) Schematic of the magnetic application conditions and sample collection times and analyses. (c) MSCs morphology cultured on the mGelatin under magnetic exposure revealed cell stretching and alignment. Scale bar: 100 µm. (d) MSCs morphology cultured on the mPVA led to heterogeneous cell organization, with the formation of necrotic aggregates and monolayer regions. Scale bar: 100 µm. Data presented as means ± SD

Upon seeding on the scaffolds, MSC cultures showed initial cell adhesion of 37–50% and 51% – 67% after 24 h, respectively, for cells cultivated on mPVA and mGelatin scaffolds (Figure 3(a-b)). Similar MSC proliferation behavior between magnetically stimulated cultures and controls was observed. The concentrations of glucose and lactate in the media, were, for all conditions, higher than 1 mM and lower than 10 mM, indicating a healthy balance without carbon source limitations and no accumulation of lactate to toxic levels (Supplementary Table S2).

The morphology of the MSCs on the mGelatin scaffolds was investigated in the presence of a magnetic field of 0.08 T (Figure 3(b-c)), highlighting the stretched and the magnetically induced orientation of the cells. Cell elongation (cell length) was statistically significantly higher in the presence of the magnetic field (120.2 ± 32 µm) than for the control condition (45.6 ± 24 µm) (Figure 3(c)). This observation can be associated to the rearrangement of the cytoskeleton as previously reported [37,38]. Confocal images revealed that, in addition to the formation of a monolayer of MSCs on the surface of the gelatin, a high number of MSCs migrated inside the scaffold, where they were still be capable to align and stretch in response to the magnetic forces. A z-stack confocal microscopy scanning of the gelatin scaffold (up to 50% of the total gel thickness) corroborated the migration effect by showing MSCs at different depth levels of the hydrogel (Supplementary Fig. S4). For the cultures on the mGelatin, the encapsulated MSCs appeared to migrate towards the borders of the scaffold, probably due to gradients of oxygen and nutrients outside the scaffold (Figure 3(b)).

For cell cultures on mPVA, the confocal imaging showed heterogeneous regions of MSC organization on the mPVA surface, with cells growing either as monolayer or aggregates loosely attached to the polymeric surface. The spontaneous formation of MSCs aggregates on the surface of the mPVA matrix (Figure 3(c)) resulted in similar diameter distribution of these 3D structures (p < 0.05) regardless of the application of the magnetic field. This suggested that the formation of these cell aggregates is probably due to the poor affinity of the cells to the matrix of the scaffold and not related to magnetic stimulation effects. Live/Dead assay performed on both the MSC cultures on mPVA, which include the 3D MSC aggregates, and on MSCs spheroids, prepared by hanging drop technique (control), evidenced the presence of a necrotic core surrounded by a thin layer of living cells. This observation suggests the difficulty of MSCs in 3D structures to get proper access to nutrients or avoid accumulation of metabolites (Supplementary Fig. S5).

### 3.4. Magnetic exposure enhances VEGF-A release from MSCs cultured on magnetic scaffolds

**Figure 4.**
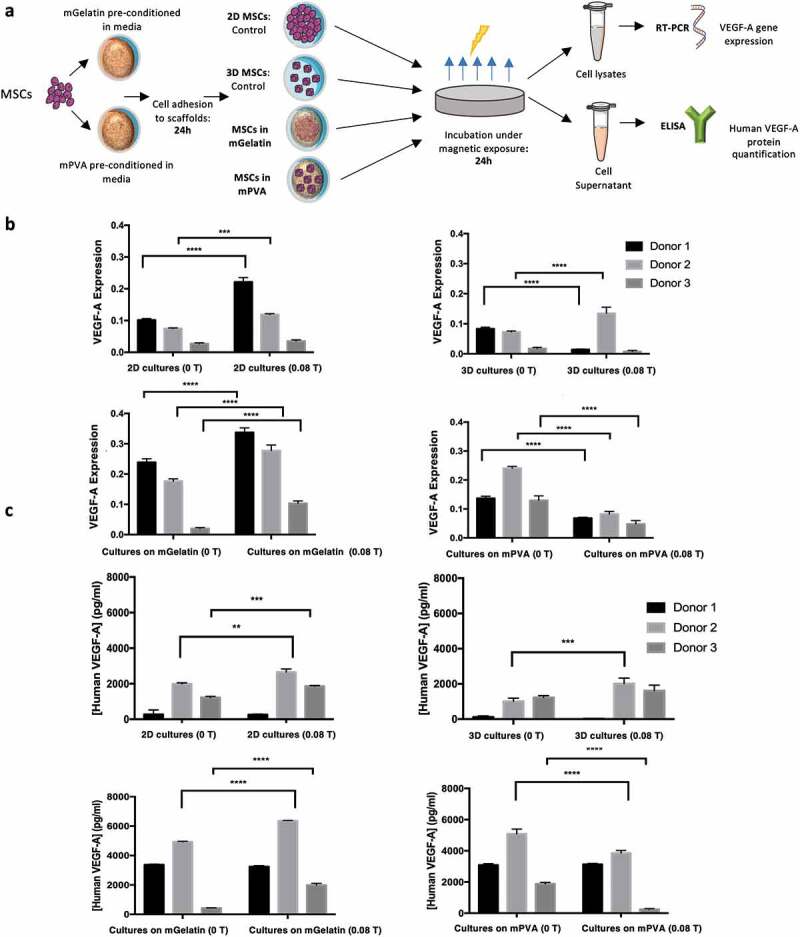
Magnetic gelatin elicits enhanced angiogenic potential whereas mPVA reveals potential for angiogenesis inhibition. (a) Schematic of the procedure involved in the measurement of the VEGF-A expression quantification in the MSCs secretome. (b) RT-PCR technique was used to assess the expression of human VEGF-A gene as 2^−ΔΔCt^. Magnetic exposure resulted in increased VEGF-A expression from MSCs in 2D cultures and in mGelatin. Reduced VEGF-A expression was obtained from mPVA. Non conclusive results were observed for the 3D cultures of MSCs. (c) ELISA specific assay for human VEGF-A was performed for protein quantification and complemented the results from RT-PCR. Statistical significance was determined using two-Way ANOVA. Data presented as means ± SD

VEGF-A mRNA expression was assessed via RT-PCR for monolayer 2D culture of MSCs (control for the mGelatin culture), 3D aggregates (control for the mPVA culture, Figure 4(a)), MSC cultures on mGelatin scaffolds and MSCs cultured on mPVA. Statistically significant increases in VEGF-A expression due to magnetic stimulation (0.08 T) were observed for the control 2D MSC monolayer, when compared to non-stimulated cultures (Figure 4(b)). VEGF-A expression of MSCs was further enhanced when those cells were cultivated on mGelatin scaffolds (scaffold doped with MNPs) under magnetic stimulation (0.08 T), in comparison with MSCs cultured either on the same scaffolds without magnetic field (0 T) or on 2D monolayer conditions (0 T and 0.08 T) (Figure 4(b)).

Interestingly, a different behavior was obtained with the MSCs cultured on mPVA, where the exposure to the magnet (0.08 T) revealed a significant decrease in the expression of VEGF-A when compared cells cultivated in absence of magnetic stimuli (0 T) (Figure 4(b)). However, for the controlled spheroids, possibly due to higher mobility freedom of these spheroids within the media, the effect of the magnetic field is not homogeneous, leading to inconclusive results. Similar VEGF-A expression profiles were observed for both the control 2D and 3D cell cultures without magnetic exposure (Figure 4(b)).

The translation of VEGF-A mRNA expression into VEGF-A protein release in the cell culture media was assessed by ELISA measurements (Figure 4(c)). The results obtained for the MSCs cultured on mGelatin as well as for the control 2D monolayer were aligned with the RT-PCR results, as the same pattern was observed with an increase verified for the conditions exposed to magnetic field. Once more, RT-PCR and ELISA results are in agreement for MSC cultures on PVA scaffolds, with inhibition of the VEGF-A expression when the scaffold is stimulated by the magnet. The magnetic effect on 3D cultures remained inconclusive in these analyses.

Overall, the results showed robustness and consistency throughout the experiments, pointing out for the interesting contrast between magnetic induction of VEGF-A release by MSCs cultures on mPVA and mGelatin, with potentially different future applications. The follow-up studies focused on the use of MSCs conditioned media to perform angiogenic functional assays.

### 3.5. *Improved HUVECs* in vitro *sprouting induced by MSCs exposure to magnetic stimulation*

**Figure 5.**
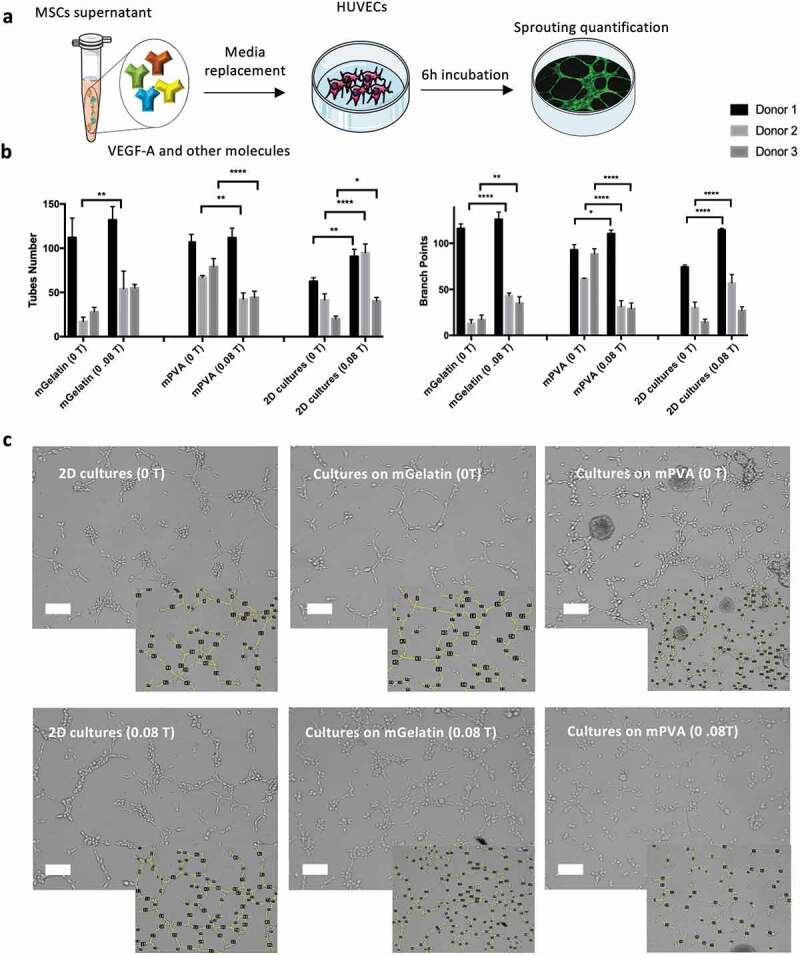
*In vitro* angiogenic sprouting of human endothelial cells (HUVECs) is enhanced by conditioned media from MSCs cultures though indirect magnetic stimulation. (a) Schematic of the procedures followed for sprouting quantification. (b) Average number of tubes, branch points and length of the tubes formed by HUVECs after 6 h of incubation with MSCs conditioned media were quantified. A significant increase in the number of tubes and branch points was observed for magnetic stimulated conditions (2D cell cultures and cell cultures on mGelatin). mPVA scaffolds induced sprouting inhibition in HUVECs cultured with the conditioned media from MSCs growing on mPVA scaffolds under magnetic exposure. (c) Representative images of the sprouting effect observed in HUVECs cultures for each condition. A higher number of ramifications was observed due to magnetic stimulation in MSCs 2D cultures and mGelatin conditions and angiogenic decrease was associated with the magnetic impact on the mPVA. Scale bar: 100 µm. Statistical significance was determined using two-Way ANOVA. Data presented as means ± SD

The complex network of the vascular system is composed of tubular structures meant to distribute nutrients, oxygen and cells to the organs. The previous determination of higher levels of VEGF-A in the media of MSCs cultivated under magnetic exposure (MSCs cultured on 2D TCP and on mGelatin-based scaffolds) suggested a positive effect of the magnetic field using MSCs to promote angiogenesis. Hence the impact of these findings on the angiogenesis process was explored through an *in vitro* study of the capacity of these VEGF-A molecules to induce the formation of tube networks in the presence of HUVECs (Figure 5(a)). The sprouting quality was quantified in terms of tube number, branch points and tube length induced by the use of conditioned media from the MSCs cultured on the scaffolds (mPVA and mGelatin) and as 2D cell monolayer on standard tissue culture TCP (control) (Figure 5(b)).

The results showed significant effect of the magnetic field on the control 2D cell monolayer and mGelatin, with higher tube numbers and branch points being formed using the conditioned media from MSCs exposed to 0.08 T. Oppositely, mPVA scaffolds coupled with the magnetic field induced a reduction of number of tubes and branch points, for most of the donors studied. Qualitatively, the impacts described can also be observed in representative images of the branching resulting from the treatment with the conditioned media (Figure 5(c)). Tube length did not provide conclusive data from any tested group possibly due to the high heterogeneity of tubes analyzed (Supplementary Fig. S6). Overall, this information can be correlated with the data obtained from mRNA expression and protein quantification.

### 3.6. Induced faster response on HUVECs wound healing and MSCs migration are related with magnetic field incidence

**Figure 6.**
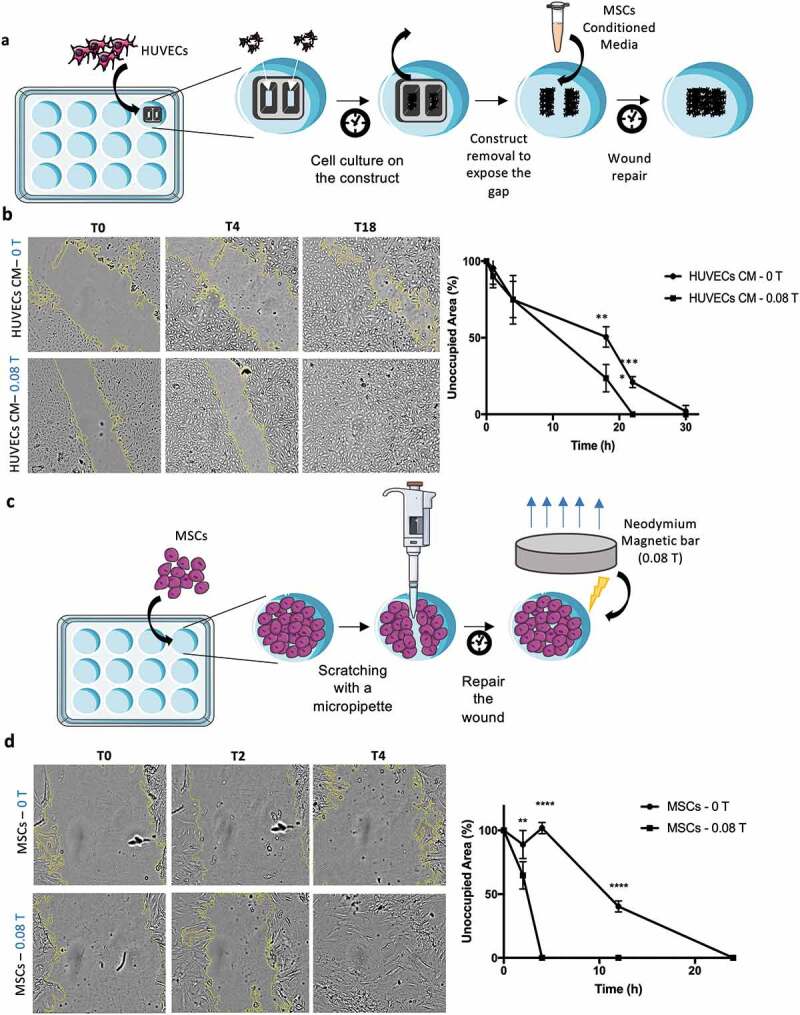
Magnetic fields application increases the velocity of wound healing by treating HUVECs with conditioned media resulting from the MSCs exposure to the magnetic source. Improvement of MSCs migration was observed through their capability to elongate and migrate according with the intensity of the magnetic field. (a) and (c) Schematic of the two methodologies used for the migration assay. (b) HUVECs were incubated for 30 h with the conditioned media (CM) from MSCs treated with magnetic field and HUVECs migration was evaluated in comparison with HUVECs treated with CM of MSCs not exposed to magnetic field. A faster repair in the scratch region was obtained for the magnetic exposure condition after 22 h. (d) A total repair of the scratch was observed after 4 h in the condition where MSCs were directly exposed to the magnetic source whereas the control condition took 24 h to reach the same effect. Statistical significance was determined using multiple t-tests. Data presented as means ± SD

Wound healing mechanisms involve several cellular processes, including cell adhesion and migration, and are mediated by several extrinsic and intrinsic cues associated with inflammatory processes. The role of MSCs and HUVECs in *in vitro* wound healing is here investigated as complementary functional assays. This assay aimed to investigate the effect of the magnetic field on the MSCs alone, without the contribution from the effects driven by the scaffolds. Therefore, the conditioned media obtained from MSCs cultured in 2D monolayer with and without magnetic stimulation were considered (Figure 6(a)). Spatial gaps were created in HUVECs confluent culture and the time periods necessary to close these gaps and for MSCs migration, in MSCs confluent cultures, were quantified. The results (Figure 6(b), Supplementary Fig. S7) revealed that the use of conditioned media from MSC cultures exposed to magnetic field (CM – 0.08 T for 4 h) induce a significant faster repair of the wound. The gap created on a HUVEC confluent layer took 22 h to be completely closed, while the controls (CM – 0 T) took 30 h to achieve the same effect. This result is in agreement with the fact that higher concentration of VEGF-A is present on the conditioned media driven by magnetic stimulated cultures.

The other aspect explored in this study focused on the possibility of mobilizing MSCs to migrate into injured tissues during healing and accelerate the process (Figure 6(c), Supplementary Fig. S8). In this study the magnet was applied directly underneath the culture of MSCs after the formation of the gap in a confluent layer of MSCs 2D monolayer. The results (Figure 6(d)) exhibited a very significant difference with a complete closure of the wounded area after 4 h for magnetic stimulated (0.08 T) MSC cultures, while no signs of wound repair were observed for the control (0 T) over the same time period. In fact, 22 h was required for a complete repair in the absence of magnetic stimulation. As noted previously, MSCs under magnetic forces tend to orientate towards the magnetic force lines which proved to be an important feature of this experiment as MSCs' ability to migrate under magnetic exposure might have contributed to the faster closure of the wound. These outcomes, both using HUVECs conditioned with MSC media after magnetic exposure or in particular the results using MSCs under magnetic stimulation, indicated a noteworthy potential for the use of magnetic fields combined with therapy MSCs for tissue repair.

### 3.7. *The magnetic effect on MSCs has the potential to prompt increased* ex vivo *vessel formation*

**Figure 7.**
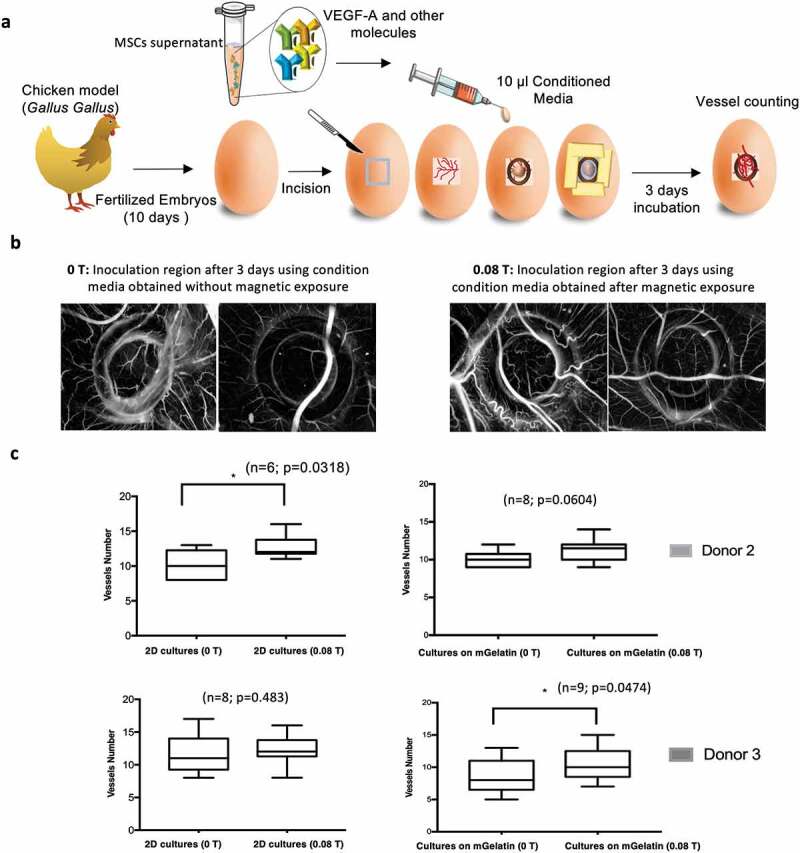
Magnetic stimulation improves *ex vivo* angiogenesis. The conditions associated with better angiogenic *in vitro* performance were selected for CAM assay. (a) Schematic of the simplified procedure involving the development of the CAM assay to assess angiogenesis phenomena allowing for an *ex vivo* validation platform of the studied conditions. (b) Images of the region of incision after 3 days of incubation with the conditioned media from MSCs exposed to magnetic stimulation (0.08 T) and the respective control condition (0 T). The determination of the number of new vessels (< 20 µm) growing towards the inoculation site during incubation period (3 days) was assessed. **c** The magnetic effect on the *ex vivo* platform was shown through statistically significant results of the MSCs control in monolayer from donor 2 where an increased number of vessels were formed due to magnetic exposure. The impact observed for the conditioned media associated with the mGelatin also showed a tendency for increased angiogenic response when exposed to magnetic field however no statistical significance was observed in these results. Statistical significance was determined using paired t-tests

Considering the promising *in vitro* results and the more stringent magnetic effects observed on the production of VEGF-A in MSCs cultured on gelatin-based scaffolds, such culture system was selected for *ex vivo* assays. This study investigates the effect of the magnetic field on the MSCs alone, without the contribution from the effects driven from the scaffolds, as well as the potential effect on the MSCs cultured in mGelatin, again with and without magnetic stimulation were. Therefore, CAM assays (Chick Chorioallantoic Membrane) were performed using fertilized chicken embryos inoculated with the conditioned media coming from MSCs cultured on mGelatin scaffolds or as 2D cell monolayers on PS culture plates (Figure 7(a), Supplementary Table S3) with and without magnetic stimulation.

After three days of inoculation, the incision area was microscopically observed detecting a difference between the controls (0 T) and the conditions treated with magnetic stimulation (0.08 T) which induced the formation of more vessels (Figure 7(b)). A comparison between the conditions revealed that, although not all the samples have reached statistical significance, the same pattern was observed for all the conditions with an increase in the number of new vessels attributed to the magnetic stimulation (Figure 7(c)). Although major conclusions are difficult to report due to the variability associated with using living embryos, collectively our findings uncovered the opportunity to explore the magnetic field as an important tool for angiogenesis improvement.

## 5. Discussion

Several studies have emphasized the importance of therapeutic angiogenesis as a valuable tool in the pathogenesis and treatment of vascular diseases by stimulating the growth of new blood vessels from pre-existing vessels [39,40]. The ultimate efficacy resides in the controlled delivery of the therapeutic proteins to the targeted tissues to actively sustain their long-term bioactivity [41]. Yet, as tissue regeneration implicates months to heal and requires persistent stimulation of growth factors, these systems are still far from representing an ideal condition. Thus, a therapeutic intervention in which the cells are non-invasively stimulated to secrete growth factors in a targeted tissue is an interesting approach, still to be developed. To overcome the aforementioned challenges, a strategy was devised in this work to remotely enhance the secretion of growth factors by MSCs using magnetic scaffolds loaded with MNPs to drive the magnetic effect on the targeted material. We successfully demonstrated that low intensity static magnetic fields can efficiently induce the release of growth factors, specifically VEGF-A, from MSCs and prompt angiogenesis *in vitro*. However, the underlying mechanism remains unclear.

Previous reports have highlighted that cells are able to communicate by transmission of electromagnetic cues and that endogenous electrical potentials appear in wounded tissues and disappear during the regeneration process, demonstrating the importance of applying the external stimuli in a specific stage of healing instead of continuously throughout the entire process [42–44]. In our experiments, magnetic stimulation was applied for a maximum of 24 h to minimize adverse effects to the cells such as oxidative stress and genotoxic effects [16]. The potentially toxic effect of longer magnetic exposure might be attributed to the situation where the spin of electrons in free radicals becomes affected leading to changes in chemical reaction kinetics and possibly altering cellular functions [45]. As a consequence, for this angiogenic strategy to be translated to an *in vivo* environment in the future, the magnetic stimulation should be applied for shorter periods of time, potentially involving cycles of stimulation to maintain cell stress and the secretion of VEGF-A.

In our study we were able to identify some effects of the static magnetic field on the MSCs. Accordingly, the proliferation rate and the plasticity of the MSCs, determined by their capability to differentiate into different cell types, do not seem influenced by the moderate intensity [44] magnetic exposure. However, we reported a magnetic impact on MSCs shape, alignment and migration, as well as on the paracrine effect associated with the secretion of molecules. Other works have explained how static magnetic fields stimulate the movement of cellular ions, increasing the consumption of oxygen by the cells and activating integrins, thus modulating cellular metabolism, clonogenic potential and cell cycle [46–48]. Consequently, beneficial effects have been observed on nerve regeneration, inflammation, united fractures and pain management [46–48]. As described, magnetic exposure modifies the shape of MSCs and promotes cell orientation. This can be justified since the magnetic field interferes with the cell key ion distributions and has been described to activate Na^+^/K^+^ channels and regulate Ca^2+^ transport, consequently affecting cell shape, size, membrane surface and distribution of cellular organelles by modulating Ca^2+^ concentration and triggering changes in the actin microdomain [49–51].

The effect of magnetic field on MSCs migration was observed in the scratch assay, leading to a faster repair of wounds created within the monolayer. Partially, this effect can be explained by the trophic and immunomodulatory properties of MSCs, which play an important role in tissue regeneration [52]. These properties identify MSCs as sensors of the inflammatory microenvironment through cell-to-cell contact and regulatory molecule secretion, including growth factors, chemokines, cytokines and extracellular vesicles [53]. Still, we observed a significantly faster response to wound healing when using magnetic stimulation. In adipose-derived stem cells, the magnetic effect also results in restoring cell polarity, interfering with different types of mechanoreceptors such as integrins. The overexpression of such molecules has been shown to mediate shear-stress and induce cell migration [50,54,55]. It was also reported that static magnetic fields enhanced the secretion of anti-inflammatory cytokines (IL-10), while controlling the secretion of pro-inflammatory cytokines (IL-6, IL-8 or TNF-alpha) to exert its anti-inflammatory properties [56].

In the present work, despite donors’ heterogeneity, we demonstrated a clear influence of the magnetic field on the secretion of VEGF-A molecule. The role of MSCs on the differentiation of endothelial progenitor cells through paracrine mechanisms involving the secretion of the cytokines (e.g. VEGF) has also been reported, with a potentially high contribution to endothelial repair and angiogenesis following ischemia, which is the focus of our work [4]. A particular study has described effective endothelial sprouting angiogenesis event using VEGF-A as a single angiogenic factor dependent on the Notch signaling in a microvessel-on-a-chip [57]. Here we also demonstrate differences in the sprouting quality of HUVECs based on the amount of VEGF-A present in the conditioned media of MSCs exposed to different scaffolds and magnetic exposure conditions.

Interestingly, our scaffolds of mPVA and mGelatin induced different cell behavior. mPVA was associated with the inhibition of the VEGF-A production from MSCs when magnetic field was applied, consequently preventing HUVECs maturation and sprouting. This denoted a probable inhibition of the VEGF pathway as demonstrated in reports targeting the inhibition of tumor cell proliferation and tumor angiogenesis [58]. Contrarily, mGelatin scaffolds increased VEGF-A signaling prompting angiogenesis phenomena. These differences are possibly related to the nature of the matrix and cells interaction with these surfaces. In comparison with synthetic polymer PVA, gelatin possesses an excellent bio-affinity and mimics the components of the extracellular matrix, providing better environment for cell attachment and growth [59]. In 3D scaffolds oxygen supply is more complex than in monolayer cultures, influenced by the chemistry and dimensions of the scaffold, cell type, density and diffusion mechanisms, with oxygen concentration decreasing from the periphery toward the center of the scaffold, creating a more hypoxic environment inside [60]. Similarly, in the bone marrow niche MSCs are also under a hypoxic condition, justifying the tendency of these bone marrow-derived cells to seek similar oxygen gradient and potentially explaining their encapsulation in the mGelatin [60]. Consequently, hypoxia conditions are known to increase VEGF-A expression, and actively affecting angiogenesis [61,62], suggesting that the higher amount of VEGF secreted in mGelatin scaffolds might be related with the MSCs encapsulation within the mGelatin. In our previous report the effect of the magnetic field on the scaffold was ascribed to mechanical structural changes and MNPs particle-particle and particle-field attraction [32]. Assuming these structural modifications impact on the encapsulated MSCs, this effect, combined with the magnetic stress induced on the cells, might be enough to elucidate the increased VEGF-A expression and secretion upon magnetic stimulation. Contrarily, MSCs poor adhesion to synthetic mPVA and the heterogeneous impact of the magnetic field over both aggregates and monolayer regions might explain the decreased secretion of VEGF-A observed.

Few reports are found in the literature concerning the application of magnetic fields to stimulate VEGF-A secretion in MSCs, which represents the novelty of this study. In other works magnetic field was used in clinical applications to increase the secretion of stem cell-derived microvesicles, known for transporting several trophic/signaling molecules in equine adipose-derived stem cells [63–65]. Still, technical challenges limit large-scale microvesicles production and their therapeutic potential [64–66].

Although positive effects of intravenous administration of angiogenic factors on tissue regeneration have been reported, the short half-life of VEGF-A *in vivo* (~30 minutes) and its off-target site toxicity represents drawbacks [67]. Our results highlighted a stronger impact of the magnetic field when applied directly to the MSCs in comparison with the indirect magnetic effect of using the conditioned media of stimulated MSCs, as observed in sprouting and wound healing assays. *Ex vivo* CAM assay might have also improved with a continuous release of growth factor to increase branching formation.

A potential clinical approach to our system would involve mGelatin as a cell support for controlled delivery of angiogenic cytokines (e.g. VEGF-A) externally mediated by the magnetic field to the injured blood vessel repair while promoting adequate vascularization (Supplementary Fig. S9).

In this work, we present a versatile technology based on magnetic responsive scaffolds for tissue engineering, that potential be used clinical translation approaches, such as non-invasive treatment of superficial skin wounds/blood vessels regeneration; incorporation in biosensors; and disease modeling, to study disease progression or drug screening, for example, in microfluidic platforms.

In summary, while the mechanism requires further evaluation and *in vivo* experiments, mPVA scaffold coupled with magnetic exposure shows great potential in cancer therapeutics by inhibiting vascularization during tumor growth, which can be a valuable tool to prevent disease progression. On the other hand, mGelatin capabilities to maximize vascularization could be explored in strategies to treat for ischemic diseases, and to promote blood vessel repair and regeneration. Those features have the potential to strongly impact in the treatment of highly prevalent vascular and cardiovascular conditions.
